# A Curiosity Estimation in Storytelling with Picture Books for Children Using Wearable Sensors †

**DOI:** 10.3390/s24134043

**Published:** 2024-06-21

**Authors:** Ayumi Ohnishi, Sayo Kosaka, Yasukazu Hama, Kaoru Saito, Tsutomu Terada

**Affiliations:** 1 Graduate School of Engineering, Kobe University, 1-1 Rokkodaicho, Nada, Kobe 657-8501, Hyogo, Japan; ohnishi@eedept.kobe-u.ac.jp; 2 Graduete School of Frontier Sciences, The University of Tokyo, 5-1-5, Kashiwanoha, Kashiwa 277-8561, Chiba, Japan; 3 Center for Spatial Information Science, The University of Tokyo, 5-1-5, Kashiwanoha, Kashiwa 277-8568, Chiba, Japan

**Keywords:** wearable computing, context recognition, curiosity estimation, child, acceleration sensor

## Abstract

Storytelling is one of the most important learning activities for children since reading aloud from a picture book stimulates children’s curiosity, emotional development, and imagination. For effective education, the procedures for storytelling activities need to be improved according to the children’s level of curiosity. However, young children are not able to complete questionnaires, making it difficult to analyze their level of interest. This paper proposes a method to estimate children’s curiosity in picture book reading activities at five levels by recognizing children’s behavior using acceleration and angular velocity sensors placed on their heads. We investigated the relationship between children’s behaviors and their levels of curiosity, listed all observed behaviors, and clarified the behavior for estimating curiosity. Furthermore, we conducted experiments using motion sensors to estimate these behaviors and confirmed that the accuracy of estimating curiosity from sensor data is approximately 72%.

## 1. Introduction

**Figure 1:**
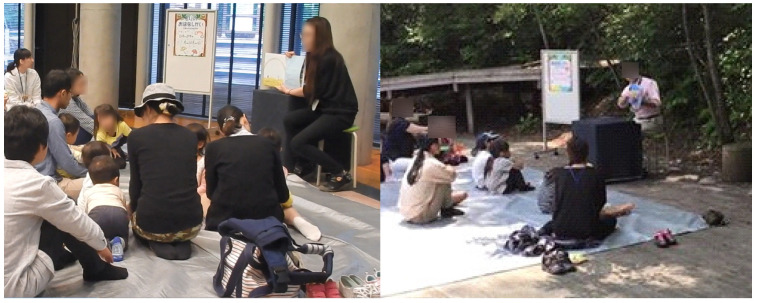
Snapshots of storytelling activities.

Storytelling activities, in which adults read aloud from picture books to children, are an important part of early childhood education. As shown in Figure 1, this activity involves adults reading picture books to children from kindergarten to early elementary school age. These activities include cases of reading a picture book for their child by parents and reading for children in a public space such as a library. Reading a picture book aloud stimulates children’s curiosities, emotional development, and imagination. It also provides an opportunity for vocabulary acquisition [1]. Childhood education has a major impact on his/her life afterward. Therefore, it is important to make storytelling activities higher quality.

Although the evaluation of activities is crucial for the reflective improvement of learning activities [2], it often becomes a bottleneck in the improvement cycle. Based on the evaluation results, the content is improved before the next implementation. There are generally two conventional methods for evaluating learning activities. The first one is a questionnaire survey. However, it is difficult for little children to answer correctly due to the problem of language capability. Since children struggle to keep concentrating for a long time, it is difficult for them to fill in a questionnaire while remembering the activity afterwards [3]. The second is a manual analysis of children’s reactions during learning by recording the video and audio and confirming the data many times. Although an evaluation is important in current storytelling activities, it is difficult to conduct at a low working cost. This improvement process cannot be performed easily. If the reaction evaluation is performed automatically using sensors, the improvement in educational activities is promoted with this improvement cycle.

Conventionally, the automatic estimation of curiosity has been attempted by eye-tracking [4] and multi-modal sensing using cameras and microphones [5,6]. However, these require expensive devices or strong restrictions on the location of users/devices. For these reasons, it is difficult to apply this method to read-aloud activities where sitting is free and many people participate.

Unlike cameras and microphones, motion sensors do not acquire data from other people because they are attached to the user’s body. Motion sensors are suitable for practical use because they can address the privacy issue of people who refuse to acquire data when they are participating in an activity. Therefore, this study takes the approach of attaching motion sensors to children’s bodies and acquiring their movements. In light of the above, the purpose of this research is to estimate the children’s curiosity level at read-aloud events based on their spontaneous responses using wearable motion sensors.

This paper aims to identify and record the characteristic reactions that can be used for automatic estimation and analyze the relationship between such reactions and the curiosity level. The proposed method estimates the level of curiosity in the activity by capturing evaluation behaviors with a motion sensor attached to the head. From an evaluation experiment in story-telling workshops, we investigated behaviors that can be recognized with high accuracy by sensors in response to high or low levels of curiosity. This paper is an extended version of the authors’ previous paper [7]. This paper improves on the previous paper by increasing the number of experiments and by summarizing the relationship between behaviors and degree of curiosity. The contributions of this paper can be summarized as the following two points:

1. This paper proposed a method for estimating the curiosity level using a motion sensor attached to the head to evaluate improvisational behavior. Four evaluation experiments in actual workshops confirmed the accuracy of the curiosity estimation.
2. This paper proposed a method to investigate the relationship between curiosity and behavior. We investigated and summarized the evaluation behaviors that can be used for reading aloud with the proposed method. In addition, we calculated the accuracy of motion recognition and summarized the evaluation behaviors that can be recognized with high accuracy by a motion sensor.

This paper is organized as follows: Section 2 introduces related research, and Section 3 describes the proposed method. Section 4 describes the experiments, and Section 5 presents the results. Based on the results, Section 6 discusses our method, and finally, Section 7 concludes the paper.

## 2. Related Research

### 2.1. Research on Motion Recognition during Conversation

Various systems have been proposed that analyze human behaviors and recognize head movements in conversation. These studies are targeted to conversations and are not analyzing the degree of curiosity. As an analysis of multiparty interaction, the sociometer [8] implemented by Choudhury et al. is a portable device consisting of a microphone, acceleration sensor, infrared module, and GPS. They aimed to visualize the social relationships of multiple persons from data obtained from gestures and conversation. The Augmented Multi-party Interaction (AMI) project aims to develop meeting browsing and analysis systems [9]. In this proposed system, the meeting corpus is recorded by using a wide range of devices, including close-talking and far-field microphones, individual and room-view video cameras, a projector, a whiteboard, and individual pens. Sumi et al. developed IMADE environments to collect various kinds of information during a conversation such as a subject’s motion, gaze, voice, and biological data [10]. Tung et al. implemented a multimodal system, which consists of a large display attached to multiple sensing devices to obtain individual speech and gazing directions [11]. Mana et al. proposed a multimodal corpus system with automatic annotation of multi-party meetings using multiple cameras and microphones. They investigated the possibility of using audio–visual cues to automatically analyze social behavior and develop a system to predict personal characteristics [12]. Okada et al. attempted to classify nonverbal patterns with gestures, e.g., utterance, head gesture, and head direction of each participant, using motion sensors and microphones [13].

Yamashita et al. attempted to evaluate activities by implementing Sounding Board [14]. This system records and shares assessments that a person expressed during conversations. However, it is difficult to use it on a daily basis because it is necessary to point a mobile device to the participant to be assessed. However, since the system requires pushing a button manually to record assessments, users may forget to push it if they concentrate on the conversation. Therefore, the automation of recording is desirable.

As in the aforementioned related studies, by accumulating everyday casual people’s evaluation gestures such as nodding or neck cranking, we can analyze these natural reactions in conversation. However, these evaluations are improvised and not recorded. If we record these casual evaluation behaviors, we may possibly be able to use it as an indicator of curiosity during learning in addition to the conventional evaluation, such as a questionnaire. Therefore, we propose a method to analyze the evaluation of children’s curiosity in storytelling by wearable sensing. In this research, we are targeting actual storytelling events; therefore, in order to take as many natural behaviors as possible, it is necessary for it to be a location-independent system. We acquire natural evaluation behaviors by using wearable sensors.

### 2.2. Research on Evaluation Methods of Children’s Learning Activities

The degree of understanding of the learning contents can be evaluated by letting the answer be selected from choices or by orally describing it [15,16]. However, our research purpose is to estimate the degree of curiosity in activities. These existing methods are difficult to evaluate. Although some studies acquire movements during learning by Kinect [17], we aim to analyze not only acquiring motions but also the degree of curiosity from such motions. Hwang et al. developed a portable system aimed at building an effective behavioral observation system for the natural settings of children’s field trips to observe the search behavior of kindergarten children on field trips [18]. However, it has not been analyzed for concrete actions or curiosity.

### 2.3. Research on Automatic Estimations of Human Intentions

Many kinds of research on the estimation of human intentions have been conducted for communication between robots and humans [5,6]. However, since these approaches are tracking individuals using cameras and microphones, these are not practical in a storytelling situation when the children’s sitting positions are not fixed. Won et al. showed a correlation between the performance of learning activities and nonverbal activities by motion analysis using Kinect. They predicted the performance level by machine learning [19]. This is different from our research in that this work did not estimate the degree of curiosity. Investigations on interest level detection of meetings [20] and spotting of excitement [21] have been conducted. These investigations target conversation-based meetings using speech recognition. However, these are not suitable for use in storytelling. Although Dodane et al. estimated a degree of interest from users’ eye movements [4], gaze analyzing devices are expensive, and their casual use for events like storytelling is not realistic.

Selene et al. analyzed children’s interest levels during computer operation from a pressure sensor value from the backrest of chairs [22]. Although this research is similar in purpose to ours in investigating the curiosity level of children, our aim is to detect curiosity in environments without chairs and computer operations.

As mentioned above, there were many kinds of research conducted for estimating curiosity and the degree of concentrations using camera images and motion sensors. However, few studies have evaluated the behaviors and curiosity of young children. To the best of the authors’ knowledge, there is no method for detecting children’s curiosity from their movements. In this study, we investigate what kind of evaluation behaviors should be measured with wearable sensors in story-telling activities to estimate the curiosity of young children automatically. Based on the result, we investigated and summarized the evaluation behaviors that can be used in story-telling activities using the proposed method. We also calculated the motion recognition accuracy and summarized the evaluation behaviors that can be recognized with high accuracy by the motion sensor.

## 3. Proposed Method

**Figure 2:**
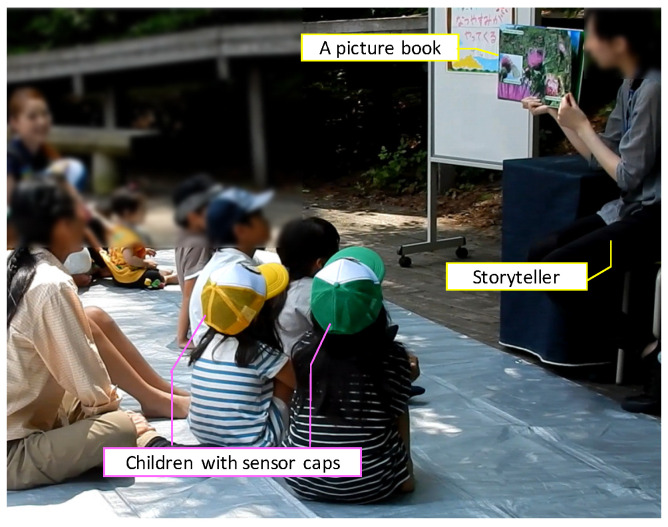
Snapshot of experiment environment. Children with sensor cap sit and gaze the direction of a picture book.

**Figure 3:**
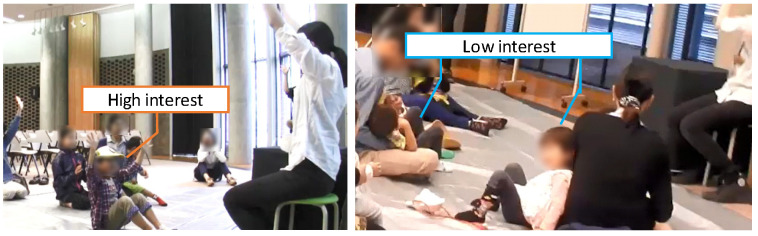
Snapshots of children who are interested or not interested in the storytelling.

In this section, we describe the requirements of the system based on the assumed environment, a proposed method of curiosity evaluation, and the procedure of the evaluation experiment. The assumed activity is the storytelling of picture books, for example, like the storytelling as shown in Figure 2. In this figure, a storyteller reads with a picture book. Children who participate in the storytelling activity sit and gaze in the direction of the picture book. During this storytelling, individual children determine their sitting positions and some infants sit on their parent’s lap. Obtaining images from the front of each child’s face with the video camera would be difficult. In Figure 3, when children are not interested in reading stories, it is assumed that they cannot stay or may go away from their sitting places.

**Figure 4:**
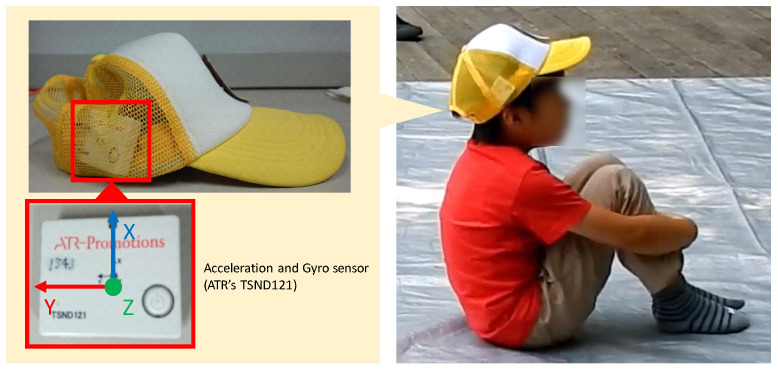
Sensor position. A motion sensor is attached to the right side of the cap.

The purpose of this paper is to estimate the curiosity level in the activity by capturing instantaneous evaluation behaviors with a motion sensor attached to the head. The configuration of the proposed system is shown in Figure 4. Children who participate in storytelling wear a cap with an ATR TSND121 sensor [23], and we acquire motion data from the acceleration and angular velocity sensor. The measurement frequency is 50 [Hz]. The measured data are wirelessly transmitted to the laptop via Bluetooth, and the laptop estimates the curiosity level.

In order to acquire the child’s natural response, it is necessary to reduce the burden on the child from wearing the sensor as much as possible so that he or she does not have to worry about the sensor. In our preliminary experiments, we tried to examine several ways to attach the sensors. As a result, when the accelerometer was attached to the cap as shown in Figure 4, the subjects did not dislike wearing the cap for a long time.

### Method for Estimating the Degree of Curiosity

To estimate the degree of curiosity for contents from child’s reactions, this study aims to examine behaviors that can be an index of curiosity during storytelling. We summarize actions associated with the degree of curiosity and that can be detected with a high level of accuracy using an acceleration and angular velocity sensor. This proposed method provides the procedure for evaluating what action can be an indicator of the degree of curiosity.

At first, children who participated in storytelling wore a cap with a sensor [23], and we acquired motion data from the acceleration and an angular velocity sensor. Video images and sounds were acquired with two video cameras to confirm the situations and to make the ground truth labels. The proposed system acquired only accelerometer data. Video and sound were acquired for the evaluation purposes. The evaluation method for estimating the degree of curiosity from motion consisted of the following four steps.

(a) We define a five-level evaluation index to quantify the levels of curiosity.
(b) Two observers manually annotate each child’s level of curiosity from the recorded video, with one observer also noting the behavior. Annotations are performed sequentially for each child.
(c) We calculate the accuracy of curiosity level estimation (defined as $C_{Ideal}$ for each action) for each action by assessing the correspondence between the action performed by the participant and the indicator in the part of the activity that the observer considered to be of high curiosity.
(d) We calculate the recognition accuracy of the behaviors observed during the narrative activity (defined as *M*) and estimate the curiosity level (defined as $C_{Actual}$) from those behaviors using the following formula:

(1) $$C_{Actual}=M\times C_{Ideal}$$

Steps (a) to (d) are described in detail below. In the designated reading story, it can be assumed that if children show interest, they predominantly direct their gaze towards the picture book and listen attentively to the narrative. Table 1 delineates the five-tiered curiosity index used for consistent assessment. the levels of curiosity are defined as follows: 5—look of curiosity; 4—listening, seems curious; 3—undecided, unable to ascertain interest from the video; 2—does not seem to be listening; and 1—obviously not interested.

In step (b), all actions observed in the video are cataloged and designated as correct answer data for curiosity, which are evaluated on a continuous five-level scale. To ensure consistency among observers, two evaluators are briefed on the evaluation criteria outlined in Table 1 prior to annotation. Given that assessments of curiosity may be subjective, it is crucial to verify their inter-rater reliability. Therefore, Cohen’s weighted kappa statistic [24,25] is utilized to confirm reproducibility between the two evaluators.

In step (c), the accuracy with which each motion reflects levels of curiosity is assessed by comparing these motions against the correctly labeled curiosity levels established in step (b). This comparison yields the ideal curiosity estimation rate ($C_{Ideal}$), assuming a 100% recognition rate for motions.

In step (d), the recognition accuracy of motions observed during the read-aloud activity is first determined. Subsequently, $C_{Ideal}$ and motion data (M) are incorporated into Equation (1) to compute the actual curiosity estimation accuracy based on sensor data, defined as the actual curiosity estimation rate ($C_{Actual}$).

By collecting and analyzing data on participants’ head acceleration and angular velocity as per steps (a) to (c), it becomes feasible to determine the curiosity estimation accuracy for each specific motion and identify which actions correlate with varying levels of curiosity. Furthermore, step (d) allows for the verification of each motion’s curiosity estimation accuracy when utilizing sensor values.

## 4. Evaluation Experiment

To identify actions that may serve as indicators of curiosity, the following experiments were conducted using the proposed method. Monthly storytelling workshops for children are held at The Mount Fuji Research Institute, Yamanashi Prefectural Government, with the primary aim of fostering curiosity about nature. The experiments discussed herein received the necessary ethical approval from the Ethical Review Committee for Research Directly Involving Human Subjects at the Graduate School of Engineering, Kobe University (approval numbers 30-22).

Evaluation experiments took place during the storytelling sessions, encompassing three different stories and a hands-on play session, lasting a total of 20 to 30 min. As depicted in Figure 1, the children were seated facing the storyteller, a staff member who presented large picture books at the front. The children wore sensor caps as shown in Figure 4. Five sensor-equipped caps were used to capture the head movements of up to five participants. Data from the sensors were downloaded after the storytelling session concluded. Additionally, with consent from all participants, including the parents, footage was captured from various angles using two video cameras to compile accurate response data. Table 2 lists the participants involved in the experiment. The subjects included 14 children from kindergarten and the lower elementary grades. The average age was approximately 6.9 years old. Data suitable for analysis were obtained from 9 of the 14 children (4 males and 5 females). The correct reaction data were annotated from the video footage using Elan software (version 4.9.4) [26]. The correlation between the motion recognition results based on acceleration and angular velocity values and the correct answers was subsequently compared and evaluated.

In this study, motion recognition accuracy was evaluated using the Weka data mining tool developed primarily at the University of Waikato [27]. The accuracy of motion recognition was assessed through 10-fold cross-validation using the C4.5 and RandomForest algorithms. The feature values for activity recognition include instantaneous values of tri-axial acceleration and angular velocity, along with their mean and variance over the past second, totaling 16 values. Variance values are adopted as feature values because they provide information on the intensity of movement, while mean values are used to convey information about posture. These features were standardized as a preprocessing step to align the scales before calculating the motion recognition accuracy. The data standardization we applied is the process of rescaling one attribute so that they have a mean value of 0 and a standard deviation of 1.

The ideal curiosity estimation rate ($C_{Ideal}$) was computed using the correct answer motion labels and the curiosity labels assigned by one evaluator at the time of each behavior. For example, if one evaluator labeled the sitting motion as curiosity level 3, and the other evaluator labeled it as curiosity level 4, the most frequently assigned curiosity level and its precision rate for the sitting motion determined the curiosity level and curiosity estimation accuracy for that action. This process was repeated for each observed motion, calculating the accuracy for both evaluator 1 and evaluator 2, and confirming whether the curiosity levels matched between observers. If the levels matched between evaluators, the values were averaged among subjects; if not, the action was deemed unsuitable for the index. This was aggregated across all participants to evaluate whether similar trends in the relationship between motion and curiosity were observed. If a participant was in an invisible position during the video recording, such as moving out of frame, that segment of data was excluded from the analysis.

## 5. Results

### 5.1. Curiosity Estimation

All actions observed in the video are cataloged in Table 3 and designated as correct answer data for curiosity, which is evaluated on a continuous five-level scale. To ensure consistency among observers, two evaluators (Evaluator 1, Evaluator 2) were briefed on the evaluation criteria outlined in Table 1 before annotation.

If a specific motion consistently indicates a high degree of curiosity across many subjects, and the actual curiosity estimation rate ($C_{Actual}$) is high, that motion can be considered a reliable indicator of curiosity. Three motions—Looking around, Looking down, and Sitting—were performed by all subjects. Among these, ‘Looking around’ was most frequently associated with varying curiosity levels, ranging dramatically from 1 to 5. Behaviors such as this ‘Looking around’, which is performed by many people but which does not have a consistent level of curiosity as evaluated by two evaluators, are less reliable as indicators of curiosity. Such behavior is calculated with a lower $C_{Actual}$. Nine actions were observed in more than 5 out of 9 participants. Among these, those that are consistent, i.e., those with high $C_{Ideal}$, are potential indicators for curiosity estimation.

Given that this evaluation experiment aims to identify actions that can serve as indicators of curiosity, all motions were listed. In practical application, when implementing the system and estimating curiosity, it is anticipated that users will select from the listed motions for recognition.

In Table 3, when the most frequently assigned curiosity level matches for Evaluators 1 and 2, a single number is recorded in the cell. If the most frequently annotated label differs between the two evaluators, it is noted as ‘3 or 4’. This indicates that Evaluator 1 is most frequently assigned a curiosity level of 3, while Evaluator 2 is assigned a level of 4. Times allocated for replacing books during story reading are considered breaks and are therefore not included in the table as they fall outside the scope of motion and curiosity analysis. The cases where the child removed the cap are excluded from the analysis, as the level of curiosity cannot be estimated.

Table 4 displays the average motion recognition accuracies for all subjects. The Random Forest algorithm achieved a higher accuracy, with an average rate of 0.92, compared to 0.81 by the C4.5 algorithm.

**Figure 5:**
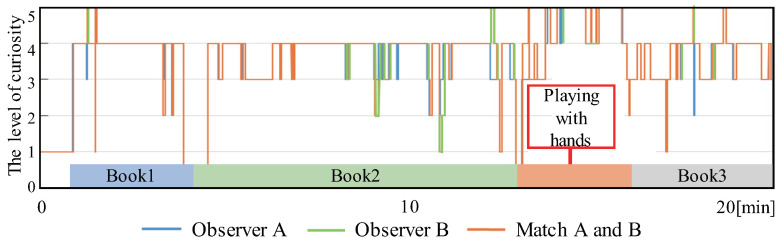
Comparison of acceleration value and degree of curiosity (Subject 0).

Figure 5 compares the evaluation of the curiosity level of two observers in time series in Subject 0. In this case, the curiosity level could be used as a curiosity indicator because the evaluater’s judgments were almost always level 5 during the period of playing with hands.

The curiosity levels are subjective and it is necessary to ascertain whether they are consistent with each other. Therefore, Cohen’s secondary weighted kappa statistics were used to confirm the reproducibility between the two evaluators. In Table 5, the reproducibility between the two evaluators was 0.96 on average. It could be said the rate was almost matched in this case.

Table 6 summarizes the behaviors from Table 3 that satisfy the two conditions described below. Condition 1 is the motion performed by two or more children. Condition 2 is that the label of curiosity that the observer mostly attached to the motion is consistent with two or more children. The average value of 17 types of the average of actual curiosity estimation rates listed in Table 6 is 0.72. In practice, not all actions in the table are used, so lower average accuracy is not a serious problem. This is because the authors assume that when developing the system, we select several target actions that are suitable for the purpose of the system from this table.

In Table 6, the smaller the value of the variance of curiosity level and the smaller the ideal curiosity estimation rate, the more consistent the index of the levels of curiosity.The variance of the curiosity level shows the closeness of the levels. The ideal curiosity estimation rate shows the rate of matches of the curiosity level. The ideal curiosity estimation rate is calculated by multiplying the motion recognition rate described in the proposed method by the ideal curiosity estimation rate and calculating the average after calculating the actual curiosity estimation rate for each individual. The largest variance among them, for example, ‘Looking around’, is 2.4 on the average curiosity level; in fact, from the results in Table 3, extreme levels, depending on the subjects, such as 1 and 5, were evaluated by observers. This indicates that “looking around” is not consistent in the relationship between subjects’ behavior and their level of curiosity. Looking back at the situation in the video, some events in the past, such as a loud noise, caused the child to look around.

If the event immediately preceding it was related to the story’s contents, since the subject obviously responded to it, the degree of curiosity was highly evaluated as 5 or 4. If the contents of the story and the looking motion were not related, it was evaluated as not interested, and the curiosity level was regarded as 1. From this result, it is necessary to distinguish whether the last event concerns the activity contents, although it expresses the extreme level of the degree of curiosity for looking around, so it is effective as an indicator. The same tendency was found for ’Looking at other people’. Regarding the ideal curiosity estimation rate, Table 6 shows that lying down and clapping, playing with hands, and talking is as low as approximately 0.7. However, each curiosity level is close to 1 or 2, or 4 or 5, etc. Since the variance value is also low as a tendency, if these motions occur, a rough degree of curiosity can be estimated with high probability.

### 5.2. Summary on the Relationship between Motions and Curiosity

The results of this study elucidate the relationship between behaviors and degrees of curiosity as detailed in Table 3, with practical indicators summarized in Table 6. Excluding the motions of looking around and looking at other people from Table 6, where the variance of curiosity levels is minimal and the ideal curiosity estimation rate is high, these indicators are considered effective for assessing degrees of curiosity. For the motions of looking around and looking at other people, it is necessary to consider the preceding events when estimating curiosity levels. In the case of nodding, its infrequent occurrence is thought to be a contributing factor to its low recognition rate. Additionally, an analysis of the video data revealed co-occurrence relationships between voice and motion, with many instances where actions in response to sounds were noted in the evaluations.

As an improvement to this evaluation method, it has been suggested that recognizing specific behaviors as indicators, analyzing their co-occurrence with other children’s behaviors, and using the degree of movement as an indicator could more effectively distinguish individuals who exhibit different behaviors from others.

## 6. Discussion

In this experiment, when actions occurred simultaneously, they were analyzed separately from singular actions because it was assumed that if they could all be identified, there would be no issue including them in the evaluation. The results showed that simultaneous actions can be challenging to recognize with motion sensors. Potential solutions include capturing these concurrent actions using cameras or increasing the number of sensors worn. While this approach can enable the recognition of complex movements, it also increases the burden and constraints on participants, necessitating careful consideration for practical implementation.

The results of this paper are limited by the number of samples and their homogeneity. To make this indicator more reliable, further validation is needed. Differences in behavior based on age and gender have been analyzed in previous studies [28]. In this experiment, although there were motions unique to individual subjects, many common behaviors were also observed among the participants. Previous research indicates that Japanese individuals tend to suppress expressions of anger [29,30]. Since this experiment focused on Japanese children, it is possible that their expressions of negative emotions, specifically disinterest, are more suppressed compared to children from other countries. To develop universal indicators, it is necessary to conduct experiments with children from various cultures for comparison. However, as there is also a need to address populations with a strong tendency towards emotional suppression, such as the Japanese, this aspect is a contribution of the current research.

In this study, the correct answer data were provided by two evaluators who watched the children’s behaviors, without directly querying the children about their interest. This approach was primarily adopted due to the difficulties associated with conducting surveys with young children as mentioned earlier. According to research by Harris et al., it is suggested that children around the age of six begin to understand the distinction between apparent emotions (such as facial expressions) and their actual internal emotions [28]. Therefore, it is possible for a child over the age of 6 to pretend to be interested without being interested. However, in the context of our storytelling sessions, there is little to gain from pretending to have no interest, so if a child appears uninterested, it is likely that they truly are not engaged. Conversely, there may be instances where a child pretends to be interested. Nevertheless, when aiming to improve storytelling activities, it is crucial to consider how to captivate children who appear uninterested. Estimating the degree of curiosity of a child who acts out a false attitude is outside the scope of this paper and is a more difficult task.

The approach using a camera that captures an overhead view has an affinity with this experiment in terms of acquiring behaviors. Therefore, we believe that the present findings are valuable for the camera approach as well. In other words, as mentioned in the introduction, it is not realistic to place an individual camera for each participant, but an approach to estimate the level of interest from an overall overhead view camera may be feasible in the future. Since the findings of this study can be used as a reference when performing such estimation, we believe that this method of estimating the degree of interest from movements will be useful when combining it with camera-based estimation.

In this study, a system to estimate the degree of interest in real time has not been constructed. We plan to implement this system in the future and realize an evaluation system based on the results of this study.

## 7. Conclusions

This paper proposed a method to estimate children’s curiosity in storytelling activities by recognizing their motions using acceleration and angular velocity sensors placed on each head. From the evaluation experiments in the actual reading activity, we investigated behaviors correlated with the degree of curiosity, and the recognition accuracy, with a motion sensor, was calculated. In the evaluation experiment carried out on storytelling activities, we acquired nine subjects’ data of kindergarten children and elementary school lower grades children, who wore a cap with an acceleration/angular velocity sensor and participated in the storytelling. We analyzed all the observed motions and listed indicators of the degree of curiosity and 17 kinds of actions that two or more children showed. We summarized these motion recognition rates and curiosity estimation rates. The average of actual curiosity estimation rates was approximately 0.72 in all subjects. Although this result is not so high on average, when we develop a system, it is assumed that we select motions for which the curiosity estimation accuracy is high from these indexes of motions. There were 5 out of 17 motions whose estimation accuracy was higher than 0.8.

In the future, we will increase the number of subjects and investigate the correspondence between motions and degree of curiosity more in detail. Also, to actually improve the activity, we will construct a system that selects and recognizes actions with high accuracy from the listed actions. It will also provide real-time feedback during the activity. This research contributes to evaluating educational activities aimed at stimulating curiosity.

## Figures and Tables

**Table 1:** Scale of five curiosity levels.

	5	4	3	2	1
Curiosity level	Look of curiosity	Listening, Seems curious	Undecided	Does not seem to be listening	Obviously not interested

**Table 2:** Breakdown of the subjects in experimentation.

The Number of Subjects	Day 1		Day 2	
	**1st Time**	**2nd Time**	**1st Time**	**2nd Time**
All subjects [persons]	5	1	5	3
Valid subjects ^1^ [persons]	0	1	5	3

^1^ Valid subjects mean full data acquired. Some children took off their caps with the sensor during the story time.

**Table 3:** Observed motions and their curiosity levels. In the case where a number is written in the cell of the table, the motion is performed by a subject, and the numerical value written in the cell is the level of curiosity evaluated by the two evaluators.

	Observed or Not, Curiosity Levels of Each Motions								
**Observed Motion**	**Sub. 0**	**Sub. 1**	**Sub. 2**	**Sub. 3**	**Sub. 4**	**Sub. 5**	**Sub. 6**	**Sub. 7**	**Sub. 8**
Looking around	1	1	2	1	5	5	1	1	5
Looking down	3	1	1	1	3 or 2	2	1	2 or 1	2
Sitting state	4	4	4	4	4	4	4	4	4
Clapping	3	1 or 2	3	-	3	3	4	4	4
Playing with hands	5	5	4 or 5	4	5	5	-	5	5
Wriggling	3	1	4	4	3	4	-	4	4
Sitting again	4	-	4 or 2	4 or 3	-	3 or 5	-	4	4
Talking	-	3 or 1	5	3 or 2	5	5	-	-	5
Looking other person	-	1	-	-	1	-	1	5	1
Finger pointing	4	5						4 or 2	
Looking back		2		1					1
Laugh					5				5
Lying down		1				2			
Nodding	4				5				
Playing with hands and talking				5					5
Sitting state and laugh				5	5				
Stretching								4 or 5	4
Touch a face							2	4	
Attention to insects				1					
Clapping and looking around								1	
Close ears				4 or 2					
Close ears and looking down				1					
Close ears and looking side				1 or 2					
Close eyes and ears				2					
Driving away insects			1						
Looking around and pointing			5						
Looking forward		1 or 5							
Looking side				2 or 4					
Mother’s movement		4							
Moving location						1			
Origami		1							
Playing with hands and looking around								5	
Scratch a head									4
Scratch arms				4					
Sneezing					3				
Stand up		1							
Stand up and laugh					4				
Talking and Laugh				5					
To tilt a head	3 or 4								
Touching a cap							2		
Touching own arm		1							
Walking		1							

**Table 4:** Motion recognition rates by acceleration/angular-velocity sensors.

	Subject No.									
	**0**	**1**	**2**	**3**	**4**	**5**	**6**	**7**	**8**	**Ave.**
C4.5	0.77	0.72	0.84	0.78	0.84	0.72	0.91	0.83	0.87	0.81
Random Forest	0.88	0.89	0.95	0.92	0.93	0.93	0.95	0.92	0.93	0.92

**Table 5:** Cohen’s weighted kappa statistics between evaluators.

Subject No.	Cohen’s Weighted Kappa Statistics
0	0.95
1	0.96
2	0.95
3	0.95
4	0.99
5	0.92
6	0.99
7	0.95
8	0.97
Ave.	0.96

**Table 6:** Summary of curiosity estimation rate.

	Ave. of Curiosity Level	Var. of Curiosity Level	Number of People	Motion Recognition Rate	Ideal Curiosity Estimation Rate	Ave. of Actual Curiosity Estimation Rate
Looking back	1.3	0.22	3	0.81	0.95	0.87
Lying down	1.5	0.25	2	0.85	0.69	0.84
Looking down	1.6	0.53	9	0.92	0.92	0.71
Looking other person	1.8	2.56	5	0.90	0.96	0.64
Looking around	2.4	3.36	9	0.72	0.90	0.58
Touching face	3.0	1.00	2	0.96	0.88	0.89
Wriggling	3.4	0.98	8	0.69	0.96	0.66
Clapping	3.4	0.24	8	0.93	0.71	0.70
Sitting again	4.0	0.00	6	0.87	0.96	0.68
Sitting state	4.0	0.10	9	0.91	0.92	0.85
Nodding	4.5	0.25	2	0.80	0.84	0.44
Finger pointing	4.5	0.25	3	0.94	0.90	0.60
Playing with hands	4.9	0.12	8	0.88	0.99	0.79
Laughing	5.0	0.00	2	0.71	0.99	0.77
Playing with hands	5.0	0.00	2	0.83	0.73	0.70
Playing with hands	5.0	0.00	2	0.83	0.73	0.70
and talking						
Sitting state and laughing	5.0	0.00	2	0.88	0.99	0.87
Talking	5.0	0.05	6	0.93	0.85	0.63

## Data Availability

Data available on request due to the privacy restriction.

## References

[1] Trelease J. (2013). The Read-Aloud Handbook.

[2] Gagne R.M., Wager W.W., Golas K.C., Keller J.M., Russell J.D. (2005). Principles of instructional Design. Perform. Improv..

[3] Punch S. (2002). Research with Children: The Same or Different from Research with Adults?. J. Child..

[4] Dodane J.B., Hirayama T., Kawashima H., Matsuyama T. Estimation of User Interest using Time Delay Features between Proactive Content Presentation and Eye Movements. Proceedings of the 3rd International Conference on Affective Computing and Intelligent Interaction and Workshops 2009 (ACII 2009).

[5] Lukac M., Kameyama M., Migranova Y. Live-feeling Communication: Multi-algorithm Approach to the Estimation of Human Intentions. Proceedings of the Conference on IEEE International Conference on Systems, Man and Cybernetics 2017 (SMC 2017).

[6] Batliner A., Steidl S., Nöth E. Associating Children’s Non-verbal and Verbal Behaviour: Body Movements, Emotions, and Laughter in a Human-robot Interaction. Proceedings of the IEEE International Conference on Acoustics, Speech and Signal Processing (ICASSP).

[7] Ohnishi A., Saito K., Terada T., Tsukamoto M. Toward Interest Estimation from Head Motion Using Wearable Sensors: A Case Study in Story Time for Children. Proceedings of the 19th International Conference on Human—Computer Interaction (HCII 2017).

[8] Choudhury T., Pentland A. Sensing and Modeling Human Networks using the Sociometer. Proceedings of the 7th IEEE International Symposium on Wearable Computers (ISWC 2003).

[9] Carletta J., Ashby S., Bourban S., Flynn M., Guillemot M., Hain T., Kadlec J., Karaiskos V., Kraaij W., Kronenthal M. The AMI Meeting Corpus: A Pre-announcement. Proceedings of the International Workshop on Machine Learning for Multimodal Interaction (ICMI-MLMI 2005).

[10] Sumi Y., Yano M., Nishida T. Analysis Environment of Conversational Structure with Nonverbal Multimodal Data. Proceedings of the International Conference on Multimodal Interfaces and the Workshop on Machine Learning for Multimodal Interaction (ICMI-MLMI 2010).

[11] Tung T., Gomez R., Kawahara T., Matsuyama T. (2014). Multiparty Interaction Understanding Using Smart Multimodal Digital Signage. IEEE Trans. Hum.-Mach. Syst..

[12] Mana N., Lepri B., Chippendale P., Cappelletti A., Pianesi F., Svaizer P., Zancanaro M. Multimodal corpus of multi-party meetings for automatic social behavior analysis and personality traits detection. Proceedings of the International Conference on Multimodal Interfaces and the 2007 Workshop on Tagging, Mining and Retrieval of Human Related Activity Information (ICMI-TMR 2007).

[13] Okada S., Bono M., Takanashi K., Sumi Y., Nitta K. Context-based Conversational Hand Gesture Classification in Narrative Interaction. Proceedings of the 15th ACM on International Conference on Multimodal Interaction (ICMI 2013).

[14] Yamashita J., Kato H., Ichimaru T., Suzuki H. Sounding Board: A Handheld Device for Mutual Assessment in Education. Proceedings of the Extended Abstracts on Human Factors in Computing Systems (CHI 2007).

[15] Wang Z., Williamson R.A., Meltzoff A.N. (2018). Preschool Physics: Using the Invisible Property of Weight in Causal Reasoning Tasks. PLoS ONE.

[16] Waismeyer A., Meltzoff A.N. (2017). Learning to Make Things Happen: Infants’ Observational Learning of Social and Physical Causal Events. J. Exp. Child Psychol..

[17] Kamizono T., Abe H., Baba K., Takano S., Murakami K. Towards Activity Recognition of Learners by Kinect. Proceedings of the IIAI 3rd International Conference on Advanced Applied Informatics (IIAIAAI 2014).

[18] Hwang I., Jang H., Park T., Choi A., Hwang C., Choi Y., Song J. Toward Delegated Observation of Kindergarten Children’s Exploratory Behaviors in Field Trips. Proceedings of the 13th international conference on Ubiquitous computing (UbiComp 2011).

[19] Won A.S., Bailenson J.N., Janssen J.H. (2014). Automatic Detection of Nonverbal Behavior Predicts Learning in Dyadic Interactions. IEEE Trans. Affect. Comput..

[20] Gatica-Perez D., McCowan L., Zhang D., Bengio S. Detecting Group Interest-level in Meetings. Proceedings of the IEEE International Conference on Acoustics Speech and Signal Processing 2005 (ICASSP 2005).

[21] Wrede B., Shriberg E. Spotting “Hot spots” in Meetings: Human Judgments and Prosodic Cues. Proceedings of the Eighth European Conference on Speech Communication and Technology.

[22] Selene M., Rosalind W.P. Automated Posture Analysis for Detecting Learner’s Interest Level. Proceedings of the Conference on IEEE Computer Vision and Pattern Recognition Workshop 2003 (CVPRW 2003).

[23] TSND121, ATR-Promotions. http://www.atr-p.com/products/TSND121.html.

[24] Cohen J. (1968). Weighted kappa: Nominal Scale Agreement with Provision for Scaled Disagreement or Partial Credit. Psychol. Bull..

[25] Fleiss J.L., Cohen J., Everitt B.S. (1969). Large Sample Standard Errors of Kappa and Weighted Kappa. Psychol. Bull..

[26] ELAN, Max Planck Institute. https://tla.mpi.nl/tools/tla-tools/elan/.

[27] Weka 3, The University of Waikato. http://www.cs.waikato.ac.nz/ml/weka/.

[28] Harris P.L., Donnelly K., Guz G.R., Pitt-Watson R. (1986). Children’s Understanding of the Distinction Between Real and Apparent Emotion. Child Dev..

[29] Lewis M., Haviland-Jones J. M., Barrett L. F. (2010). The Emergence of Human Emotions. Handbook of Emotions.

[30] Zahn-Waxler C., Friedman R.J., Cole P.M., Mizuta I., Hiruma N. (1996). Japanese and United States Preschool Children’s Responses to Conflict and Distress. J. Child Dev..

